# Data regarding the sensibility to proteolysis of a natural apolipoprotein A-I mutant.

**DOI:** 10.1016/j.dib.2020.105960

**Published:** 2020-07-03

**Authors:** Gisela M. Gaddi, Romina A. Gisonno, Silvana A. Rosú, M. Fernanda Cortez, Gabriela S. Finarelli, Nahuel A. Ramella, M. Alejandra Tricerri

**Affiliations:** a Instituto de Investigaciones Bioquímicas de La Plata (INIBIOLP), CONICET, La Plata, Buenos Aires, Argentina; b Facultad de Ciencias Médicas, Universidad Nacional de La Plata, Calle 60 y 120. La Plata, Buenos Aires, Argentina

**Keywords:** Apolipoprotein A-I -partial proteolysis analysis-protein structure and catabolism-protein flexibility

## Abstract

The article shows dataset of the proteolysis of a natural variant of apolipoprotein A-I (apoA-I) with a substitution of a leucine by and arginine in position 60 (L60R), in comparison with the protein with the native sequence (Wt). This information demonstrates the potential of *in vitro* partial proteolysis experiments as it may be applicable to different approaches in the biophysical field. We have analyzed by different electrophoresis techniques apoA-I variants, quantified the degree of proteolysis after staining and compared the proteolysis efficiency with the computed cleavage patterns. The data shown here clearly strengthen the usefulness of this approach to test protein flexibility, as it may be attained with enzymes which are not expected to modify *in vivo* this protein but have a well-known digestion pattern. In addition it is appropriate for evaluating protein catabolism, as it is exemplified here by the evidence with metalloproteinase 12 (MMP-12), which is a physiological protease that may elicit the pro-inflammatory processing of this variant within the lesions. We support the work “Structural analysis of a natural apolipoprotein A-I variant (L60R) associated with amyloidosis” (Gaddi, et al., 2020), gaining insights on protein folding from a characterization by proteolysis analysis [1].

## Specifications Table

**Table:** 

**Subject**	Biochemistry and Biophysics
**Specific subject area**	Protein structure analysis
**Type of data**	Tables (2)Figures (4)Excel doc (1 Supl)
**How data were acquired**	Data include experimental designs of proteolysis susceptibility, involving analysis of proteins treated with well-known proteases, the obtained digestion products developed by polyacrylamide gel electrophoresis, stained and followed by quantification with the Image J 1.51 j8 Software. These data were compared with the peptide pattern predicted for complete proteolysis, by using the Peptide Mass software, available through the ExPASy World Wide Web server
**Data format**	Data are presented asText in word formatFigures as TiffRaw in excelAnalyzed
**Parameters for data collection**	Data are taken from triplicates of experimental data, proteins are recombinant from bacterial expression systems
**Description of data collection**	Data are built from the quantification of the bands obtained from the digestion pattern, the comparison among two related protein variants and the prediction from expected peptides by using available predictors.
**Data source location**	Institution: Instituto de Investigaciones Bioquímicas de La Plata (INIBIOLP)City: La Plata, Buenos AiresCountry: Argentina
**Data accessibility**	With the article
**Related research article**	G.M. Gaddi, R.A. Gisonno, S. A. Rosú, L.M. Curto, E.D. Prieto, G.R. Schinella, G.S. Finarelli, M.F. Cortez, L. Bauzá, E.E. Elías, N.A. Ramella, M.A. Tricerri. Structural analysis of a natural apolipoprotein A-I variant (L60R) associated with amyloidosis Arch Biochem Biophys 685 (2020) 108,347. doi: 10.1016/j.abb.2020.108347

## Value of the data

• Data shows a powerful design to compare arrangement, conformation and flexibility of proteins as it offers an inexpensive tool to support biophysical approaches.
• These data may benefit to the extended field of protein studies as it may give information either on structure or on physiological catabolism.
• This approach may be combined with mass spectrometry and bioinformatics analysis, and adapted to different proteins as a function of the information that is available on molecular weight or sequence.

## 1 Data description

**Fig 1:**
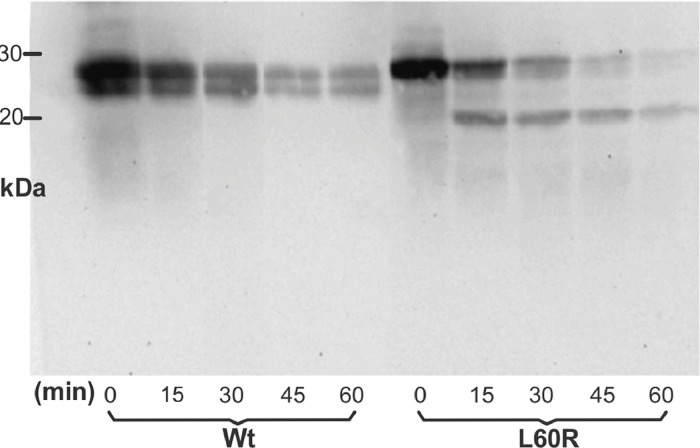
**Characterization of the L60R proteolysis fragment** Trypsin-treated proteins were separated on a 12–24% SDS gradient gel electrophoresis (SDS-PAGGE), and developed by western blotting against polyclonal apoA-I antibody. SDS LMW standard (GE, Healthcare, UK) was used as molecular weight markers.

Wt and L60R were incubated at increasing times with trypsin and reactions stopped after different time periods (0, 15, 30, 45 and 60 min). The digestion products were analyzed by gradient gel electrophoresis (SDS-PAGGE) and developed by western blot using a specific antibody against apoA-I (Fig. 1).

In order to determine whether the mutation may modify the digestion pattern, the computed mass of the peptides that could yield the proteolysis of the first 100 amino acids of apoA-I was analyzed (Table 1). If complete, trypsin treatment should result in 14 peptides from 2 to 17 amino acid length. The replacement of the L by an R in the variant is predicted to split the dipeptide 60–61 into two single amino acids, thus incorporating a cleavage site.

**Table 1:** Computed cleavage pattern from tryptic digestion of the first 100 amino acids of apoA-I variants.

	**Wt (1–100)**		**L60R (1–100)**	
	**Peptide mass**^a^	**Amino acid**^b^**residues**	**Peptide mass**^a^	**Amino acid**^b^**residues**
1	1932.9337	62–77	1932.9337	62–77
2	1612.7853	46–59	1612.7853	46–59
3	1400.6692	28–40	1400.6692	28–40
4	1235.6881	13–23	1235.6881	13–23
5	1226.5436	1–10	1226.5436	1–10
6	732.3774	89–94	732.3774	89–94
7	704.3573	78–83	704.3573	78–83
8	622.2865	84–88	622.2865	84–88
9	615.3824	41–45	615.3824	41–45
10	506.2609	97–100	506.2609	97–100
11	434.1994	24–27	434.1994	24–27
12	288.2030	60–61	–	–
13	246.1812	11–12	246.1812	11–12
14	218.1499	95–96	218.1499	95–96
15	–	–	175.1189	60–60
16	–	–	175.1189	61–61
				

a Monoisotopic peptide masses given as [*M* + *H*]^+^calculated from [12].

b Occurring amino acid residues.

**Fig 2:**
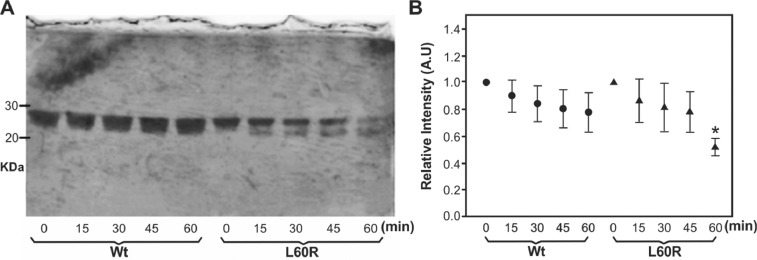
**Characterization of L60R proteolysis mediated by chymotrypsin**. A) A molar ratio of 5000: 1 apoA-I to enzyme was incubated in Tris 20 mM pH 7.4 buffer at 37 °C. Reactions were stopped after 0, 15, 30, 45 and 60 min by addition of buffer containing 0.1% SDS following by 2-min boiling. Samples developed by the silver stain were loaded and resolved through SDS-PAGE. On the left, bands corresponding to the migration of the carbonic anhydrase (30 kDa) and trypsin inhibitor (20 kDa) were labeled to use as reference. B) Efficiency of the proteolysis was estimated by quantifying intensity of the band remaining within the original molecular weight (28 kDa) and normalized to the band intensity at time= 0 for each protein. Circles and triangles correspond to Wt and L60R respectively (* represents *p*< 0.05 with respect to the same time in the Wt) by using Student's test of triplicates.

Partial proteolysis mediated by chymotrypsin was also analyzed in the same trend. Together with trypsin, this enzyme is a potent tool for structural studies involving mass spectrometry and cross-linking [2], as the cleavage pattern is well-defined (it selectively hydrolyzes peptide bonds on the C-terminal side of tyrosine, phenylalanine, tryptophan, and leucine) [3]. Partial digestion of Wt and L60R is shown in Fig. 2. Raw data of the performed analysis is shown as Supl. File 1

Again, we analyzed the peptides computed if complete proteolysis occurs of Wt (1–100) and L60R (1–100) (Table 2). Interestingly, and in spite of the higher susceptibility, the addition of R (by substitution of L) removed the recognition site which cleaves the hexapeptide 58–64 into 58–60 and 61–64 in the Wt.

**Table 2:** Computed cleavage pattern from digestion of the first 100 amino acids of apoA-I variants mediated by chymotripsin.

	**Wt**		**L60R**	
	**Peptide mass**^a^	**Amino acid**^b^**residues**	**Peptide mass**^a^	**Amino acid**^b^**residues**
1	1190.6415	91–100	1190.6415	91–100
2	955.4156	1–8	955.4156	1–8
3	–	–	916.5323	58–64
4	840.3846	23–29	840.3846	23–29
5	805.3938	76–82	805.3938	76–82
6	777.3777	65–71	777.3777	65–71
7	756.3410	51–57	756.3410	51–57
8	745.4202	9–14	745.4202	9–14
9	563.2606	83–86	563.2606	83–86
10	545.3042	61–64		
11	480.2453	30–33	480.2453	30–33
12	476.2351	34–38	476.2351	34–38
13	462.2558	87–90	462.2558	87–90
14	453.2344	15–18	453.2344	15–18
15	445.2769	39–42	445.2769	39–42
16	445.2656	19–22	445.2656	19–22
17	434.1670	48–50	434.1670	48–50
18	361.1718	73–75	361.1718	73–75
19	347.2289	58–60		
20	260.1968	45–46	260.1968	45–46
21	246.1448	43–44	246.1448	43–44
22	205.0971	72–72	205.0971	72–72
23	132.1019	47–47	132.1019	47–47

a Monoisotopic peptide masses given as [*M* + *H*]^+^calculated from [12].

b Occurring amino acid residues.

**Fig 3:**
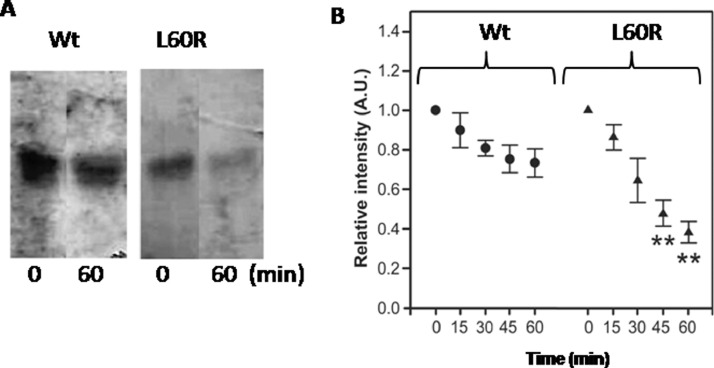
**Time-dependent proteolysis of Wt and L60R induced by human MMP-12:** Proteins were incubated at 0.3 mg/mL in Tris 20 mM buffer pH 7.4 with MMP-12 at molar ratio of 500:1 apoA-I variants to enzyme respectively. After different time periods, reactions were stopped by the addition of sample running buffer and two min boiling. Samples were run through a SDS-PAGE (16%) and developed by silver staining. A) Black/white representation of the initial (0) and final (60 min) incubation times. B) Intensity remaining with the monomeric molecular weight (28 kDa) after MMP-12 treatment was quantified by the Image Quant software and normalized to the intensity of the band at time= 0 for each protein. Circles and triangles correspond to Wt and L60R respectively (** represents *P*< 0.001 with respect to the same time in the Wt).

Finally, apoA-I proteolysis mediated by metalloproteinase-12 (MMP-12) was tested and the efficiency estimated by SDS-PAGE (Fig. 3). Raw data for this analysis is also included in Supl. File 1

As a difference with the previous enzymes (which are not expected to participate in physiological roles involving apoA-I catabolism), MMP-12 is highly activated in leukocytes [4], and seems to play, among other functions a detrimental role eliciting atherosclerotic lesion growth and increasing the susceptibility to rupture [5]. The existence of dysfunctional apoA-I in atherosclerotic plaques may suggest that it could be substrate of this or other pro inflammatory enzymes [6]. Unluckily, the proteolysis pattern is not possible to estimate as, even though it is proposed to cleave after alanine, valine, leucine, isoleucine, serine and threonine, the efficiency may vary on different peptides lengths [7].

## 2 Experimental design, materials and methods

### 2.1 Materials

Sodium dodecyl sulfate (SDS), guanidine hydrochloride (GdmCl), enzymes (trypsin, chymotrypsin and matrix metalloproteinase-12 (MMP-12, Catalytic Domain)), acrylamide and bis acrylamide, tetramethylethylenediamine (TEMED), luminol and coumaric acid were purchased from Sigma-Aldrich (St Louis, MO); IMAC Sepharose 6 Fast Flow Resin was acquired from GE Healthcare Bio-Sciences (AB, Uppsala, Sweden). Isopropyl-β-d-thiogalactoside (IPTG) was purchased from Thermo Scientific (Waltham, MA). Yeast extract and tryptein were purchased from Brittania (Arg). All other reagents were acquired with the highest analytical grade.

### 2.2 Experimental

#### 2.2.1 Protein variants purification

**Fig 4:**
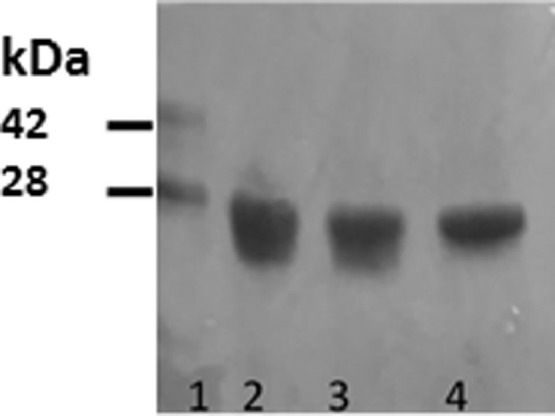
**Characterization of protein purity.** SDS-PAGE (16%) stained with Coomasie Blue (in black/white scale). Lane 1, Standard marker containing Wt apoA-I with and without the His-Tag (marks 42 and 28 kDa respectively). Lanes 2 to 4, purified fractions of Wt apoA-I after the elution through IMAC Sepharose nickel affinity columns (GE Healthcare Bio-Sciences (AB, Uppsala, Sweden)).

A Wt apoA-I cDNA template inserted into a pET-30 plasmid (Novagen, Madison, WI), was used as a template to obtain protein fused to a His-Tag peptide directly upstream of the N-terminus as previously described [8]. By using this template, the pro-amyloid mutant L60R was generated by the Quickchange method (Stratagene, La Jolla, CA) [9][10]. This setup allowed protein isolation from *E. coli* lisates by nickel affinity columns. The His-Tag was further removed by chemical cleavage [11], followed by a second metal affinity chromatography step to separate the final pure protein fraction. If required, proteins may be dialyzed against Tris 20 mM pH 7.4 (Tris buffer) and eluted through the same steps along the affinity column to obtain high quality of pure protein (Fig. 4).

#### 2.2.2 Partial degradation by proteolysis

In order to compare the influence of the substitution of a L by a R on the protein conformation, different enzymes were tested under conditions (molar ratios and time incubations), where proteolysis was not complete, thus allowing a comparison with the Wt. Variants were diluted to 0.3 mg/mL in Tris buffer, and incubated at 37 °C with mild agitation in the presence of trypsin, chymotrypsin or MMP-12, at molar ratios apoA-I variants to enzyme of 1000:1, 5000:1 and 500:1 respectively. After 0, 15, 30, 45 and 60 min, samples were combined with an appropriate amount of running buffer (containing 0.1% SDS) and heated in boiling water for 2 min. Following, variants incubated with trypsin were resolved by a gradient gel electrophoresis (12–24%) with SDS (SDS-PAGGE), and developed by western blotting using a polyclonal antibody against apoA-I [8]. Chymotripsin and MMP-12-treated samples were resolved by 16% SDS-PAGE, and developed by silver staining. The associated intensity of the remaining protein within the monomer molecular weight was quantified with the Image J 1.51 j8 software. Statistical differences were determined by the Student´s test analysis of triplicates of the samples treated under identical conditions in at least three different experiments. The relative intensity of L60R bands were compared with the same incubation time of Wt and normalized to the band intensity at time= 0. Significance is shown in each figure.

#### 2.2.3 Other analytical methods

To predict whether the proteolysis pattern could be modified by the substitution of a L by a R, the software Peptide Mass, available through the ExPASy World Wide Web server [12] was run through the first 100 residues of Wt and L60R to compute the masses of the generated peptides following trypsin or chymotripsin treatment. No missed cleavage or post-translational modifications were allowed.

Protein content was quantified by the Bradford technique [13] or by absorbance from the estimation of the extinction coefficient (32,430 M^−1^cm^−1^at 280 nm) as determined in a Bio-Rad spectrophotometer (Hercules, CA).

## Declaration of Competing Interest

The authors declare that they have no known competing financial interests or personal relationships which have, or could be perceived to have, influenced the work reported in this article.

## Appendix A Supplementary materials

Supplementary Data S1 Supplementary Raw Research Data. This is open data under the CC BY license http://creativecommons.org/licenses/by/4.0/ Supplementary Data S1

## References

[1] Gaddi G.M., Gisonno R.A., Rosú S.A., Curto L.M., Prieto E.D., Schinella G.R., Finarelli G.S., Cortez M.F., Bauzá L., Elías E.E., Ramella N.A., Tricerri M.A. (2020). Structural analysis of a natural apolipoprotein A-I variant (L60R) associated with amyloidosis. Arch. Biochem. Biophys..

[2] Giansanti P., Tsiatsiani L., Low T.Y., Heck A.J.R. (2016). Six alternative proteases for mass spectrometry-based proteomics beyond trypsin. Nat. Protoc..

[3] Wang X., Codreanu S.G., Wen B., Li K., Chambers M.C., Liebler D.C., Zhang B. (2018). Detection of proteome diversity resulted from alternative splicing is limited by Trypsin cleavage specificity. Mol. Cell. Proteom..

[4] Lindstedt L., Saarinen J., Kalkkinen N., Welgus H., Kovanen P.T. (1999). Matrix metalloproteinases-3, -7, and -12, but not -9, reduce high density lipoprotein-induced cholesterol efflux from human macrophage foam cells by truncation of the carboxyl terminus of apolipoprotein A-I. Parallel losses of pre-β particles and the high. J. Biol. Chem..

[5] Johnson J.L., George S.J., Newby A.C., Jackson C.L. (2005). Divergent effects of matrix metalloproteinases 3, 7, 9, and 12 on atherosclerotic plaque stability in mouse brachiocephalic arteries. Proc. Natl. Acad. Sci. USA.

[6] Huang Y., Didonato J.A., Levison B.S., Schmitt D., Li L., Wu Y., Buffa J., Kim T., Gerstenecker G.S., Gu X., Kadiyala C.S., Wang Z., Culley M.K., Hazen J.E., Didonato A.J., Fu X., Berisha S.Z., Peng D., Nguyen T.T., Liang S., Chuang C.-C.C., Cho L., Plow E.F., Fox P.L., Gogonea V., Tang W.H.W.W., Parks J.S., Fisher E.A., Smith J.D., Hazen S.L. (2014). An abundant dysfunctional apolipoprotein A1 in human atheroma. Nat. Med..

[7] Wenzel H.R., Tschesche H. (1981). Cleavage of peptide-4-nitroanilide substrates with varying chain length by human leukocyte elastase. Hoppe Seylers Z. Physiol. Chem..

[8] Ramella N.A., Rimoldi O.J., Prieto E.D., Schinella G.R., Sanchez S.A., Jaureguiberry M.E., Vela M.S., Ferreira S.T., Tricerri M.A. (2011). Human apolipoprotein A-I-derived amyloid: its association with atherosclerosis. PLoS One.

[9] Ramella N.A., Schinella G.R., Ferreira S.T., Prieto E.D., Vela M..., Ríos J.L., Tricerri M.A., Rimoldi O.J. (2012). Human apolipoprotein A-I natural variants: molecular mechanisms underlying amyloidogenic propensity. PLoS One.

[10] Rosú S.A., Rimoldi O.J., Prieto E.D., Curto L.M., Delfino J.M., Ramella N.A., Tricerri M.A. (2015). Amyloidogenic propensity of a natural variant of human apolipoprotein A-I: stability and interaction with ligands. PLoS One.

[11] Ryan R.O., Forte T.M., Oda M.N. (2003). Optimized bacterial expression of human apolipoprotein A-I. Protein Expr. Purif..

[12] Gasteiger E., Gattiker A., Hoogland C., Ivanyi I., Appel R.D., Bairoch A. (2003). ExPASy: the proteomics server for in-depth protein knowledge and analysis. Nucl. Acids Res..

[13] Bradford M.M. (1976). A rapid and sensitive method for the quantitation of microgram quantities utilizing the principle of dye-binding assay. Anal. Biochem..

